# Diagnostic challenges presented by women with anorexia nervosa and elevated rates of autistic traits

**DOI:** 10.1192/j.eurpsy.2021.1852

**Published:** 2021-08-13

**Authors:** D. Jeremias, C. Laginhas, D. Rodrigues, A. Moura

**Affiliations:** Psychiatry Department, Ocidental Lisbon Hospital Center, Lisboa, Portugal

**Keywords:** anorexia nervosa, autism, comorbidity, females

## Abstract

**Introduction:**

The link between autism spectrum disorder (ASD) and anorexia nervosa (AN) firstly emerged in the 80’s. Given the overlap in behavioural and cognitive features between these two seemingly different disorders, AN has been hypothesized to be a female phenotype of ASD.

**Objectives:**

This report aims to describe a clinical case of an anorexic female patient diagnosed later in life with ASD, while presenting a bibliographic review on the subject.

**Methods:**

After gaining consent, detailed information about the case history was collected and medical records were analysed and reviewed. A non-systematic literary review was performed on the Pubmed and Cochrane databases using the key words “anorexia nervosa”, “females”, “comorbidity” and “autism spectrum disorder”.

**Results:**

The current case report is of a 28-year-old female, whose extremely low body weight and complete food refusal for three days prompted her first hospitalization in a psychiatric unit with the admission diagnosis of anorexia nervosa. However, long-term impairments in social interaction and flexibility, emotional difficulties and sensory processing overload were acknowledged and the primary diagnosis of ASD was then considered.

**Conclusions:**

As illustrated in this case, the diagnosis of ASD should always be considered in females with eating disorders, in particular AN, regardless of age. As this neurodevelopmental condition appears to present differently in females, they also seem more likely to go underdiagnosed. Also, due to poorer treatment outcomes in females with both ASD and AN, the importance of developing a specialized approach and prompt referral of these patients is highlighted.

**Disclosure:**

No significant relationships.

